# Insights into Kinetics and Thermodynamics for Adsorption Methylene Blue Using Ecofriendly Zeolites Materials

**DOI:** 10.1021/acsomega.4c11718

**Published:** 2025-05-15

**Authors:** Mateus Gonçalves dos Santos, Lucas Destefani Paquini, Paulo Henrique Leite Quintela, Luciene Paula Roberto Profeti, Damaris Guimarães

**Affiliations:** † Postgraduate program in Chemical Engineering (PPEQ), 680695 Universidade Federal do Espírito Santo, Alto Universitário, s/n., Alegre, ES 29500-000, Brazil; ‡ Laboratório de Pesquisa e Desenvolvimento em Eletroquímica (LPDE), Universidade Federal do Espírito Santo, Campus Goiabeiras, Av. Fernando Ferrari, Vitória, ES 29075-910, Brazil; § Postgraduate program in Chemical Engineering, 425924 Universidade Federal de Sergipe (UFS), São Cristóvão, SE 49100-000, Brazil

**Keywords:** sustainability, wastewater treatment, adsorption, zeotypes

## Abstract

Different materials have been used as adsorbents for removing micropollutants from industrial effluents, with vegetal-derived activated carbon being widely reported for dye removal, especially methylene blue (MB). However, its high cost and environmental concerns have driven the search for alternative adsorbents. Beyond developing new materials, understanding the interaction mechanisms between adsorbents and adsorbates is crucial. In this investigation, sodium (NaZ) and protonic (HZ) zeotypes were synthesized using diatomaceous earth (DE) residue as a silicon source and applied to MB dye adsorption. Batch experiments investigated adsorption rates, mechanisms, and thermodynamic spontaneity. The results showed rapid adsorption kinetics, with equilibrium achieved in about 5 min, following the Avrami model. The Weber and Morris model highlighted that the boundary layer significantly affects film diffusion and intraparticle diffusion. The adsorption process reached equilibrium governed by the Freundlich model, with favorable adsorption behavior for all adsorbents (nF values between 1 and 10). Additionally, the enthalpy values were found to be +39.66, + 5.70, and +21.79 kJ mol ^–1^ for NaZ, HZ, and DE, respectively. This was accompanied by a decrease in Gibbs free energy with a progressive increase in temperature, indicating a more spontaneous process at higher temperatures. These results suggest that the adsorption of MB onto the synthesized zeotypes is efficient, with fast kinetics and thermodynamic favorability. The zeotypes synthesized from diatomaceous earth exhibit promising characteristics for potential large-scale applications in wastewater treatment.

## Graphical abstract

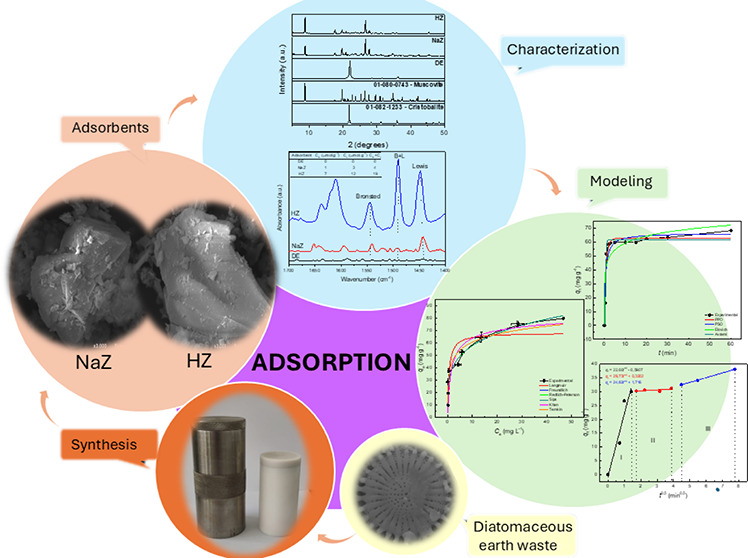

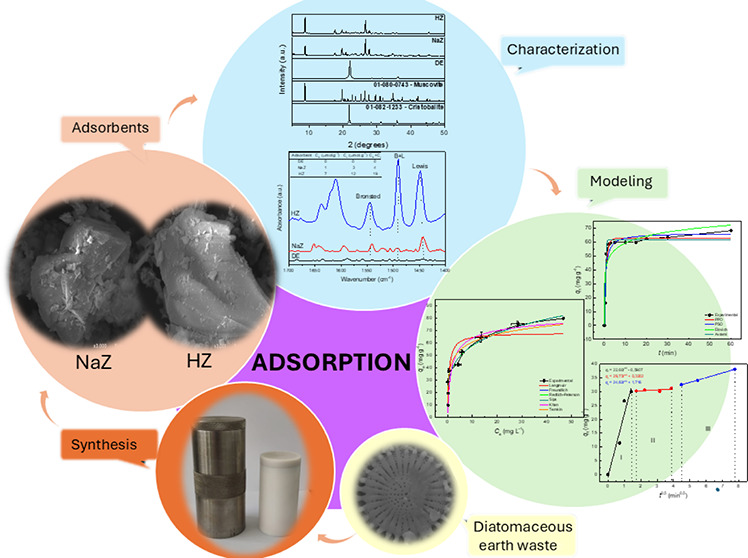

## 1 Introduction

The presence of contaminants originating from anthropogenic activities, such as agricultural, industrial, and mining waste, has caused drastic consequences for the planet.^1^ When generated and improperly disposed of, such waste can be found in water, soil, and air, posing serious risks to human life and the environment.^2^ Among the pollutants is Methylene Blue (MB), a textile dye classified as toxic.^3^

Human contact with wastewater contaminated with MB can lead to serious health issues, such as increased heart rate, cyanosis, tissue necrosis, vomiting, formation of Heinz bodies, among other diseases, due to its carcinogenic, mutagenic, and teratogenic potential.^2^ Methylene Blue when applied in the dyeing of fabrics generates a large volume of effluents, which are also contaminated with other chemical substances used in these processes, such as additives, electrolytes, toxic metals, surfactants, and pH adjusters.^4,5^

Due to its chemical stability, high solubility, and resistance to biodegradation, Methylene Blue is difficult to remove using conventional wastewater treatment methods, such as coagulation, flocculation, membrane filtration, and biological processes.^1^ Singularly, coagulation proves incapable of aggregating dye particles, particularly cationic ones, such as acridine orange and malachite green.^6^ Other drawbacks of this process include the generation of large volumes of toxic sludge, the reappearance of color in subsequent stages due to oxidation, and the increase in total dissolved solids content.^7,8^

Among the available technologies for dye removal, most have limitations that render them inefficient, such as the accumulation of toxic sludge, excessive use of chemicals, and incomplete removal of dyes.^9^ Remediation technologies associated with dye removal in textile effluents should be economically viable and capable of rapidly retaining large quantities of pollutants, without generating secondary pollution or generating as little as possible. As an alternative, the literature frequently reports the combination of different conventional wastewater treatment methods with advanced techniques to achieve better results.^7,10^ Among these, adsorption stands out due to its low generation of toxic intermediates, high removal capacity, low cost among the most efficient techniques, and potential for material reuse.^11^ Moreover, because it is highly efficient, it enables the treated effluent to meet environmental standards. Additionally, alternative adsorbents derived from agro-industrial waste can be applied in adsorption processes.^12–14^

In Wastewater Treatment Plants (WWTPs), the conventional adsorbent utilized is activated carbon, which, along with ion exchange resins, possesses high porosity and surface area—fundamental properties for effective adsorbents. However, while activated carbon can be derived from plant materials, its production may contribute to deforestation if not sourced from waste materials. Additionally, it exhibits certain characteristics that impede its efficient application in the removal of textile dyes from aqueous media. These characteristics include hygroscopicity, hydrophobicity, pore obstruction, and low thermal stability, which hinder its thermal regeneration.^15,16^

Although alternative adsorbents such as carbon-based materials, zeolites, and graphene nanoplates have been extensively studied, cost remains a significant limiting factor. In this context, adsorbents derived from agricultural waste emerge as promising alternatives due to their abundance, economic viability among the most efficient techniques, and potential for reuse according to their interaction with the adsorbate.^17–20^

In line with sustainable development, adsorbents derived from industrial and agricultural waste have also emerged, known as next-generation adsorbents, such as zeotypes synthesized from coal gangue,^21^ zeolite/hydrated metal oxide from coal fly ash,^22^ in addition to activated carbon derived from waste such as Nauclea diderrichii,^23^ avocado peel from the species Persea americana^24^ peel of Citrus limetta,^25^ have gained prominence in recent times. However, although activated carbons derived from agricultural waste contribute to the circular economy and exhibit physicochemical properties and affinity for various adsorbates, such as organic materials, inorganic substances, ions, and toxic metals,^26^ they still exhibit limitations like conventional activated carbon.

Currently, in WWTPs focused on the removal of textile dyes through adsorption, the most used adsorbents are zeolites, activated carbon, graphene oxide, and polymeric materials.^27,28^ Zeolites, crystalline aluminosilicates formed by aluminum and silicon atoms linked together by oxygen atoms, have gained prominence due to their textural properties, uniform microporosity, controllable acidity, shape selectivity, chemical and thermal stability, and recyclability, which are ideal properties for applications as adsorbents.^29^

Due to these properties, the use of zeolites in various fields has increased, as has the search for new structures. Crystalline structures of the zeolitic type, synthesized with tetrahedrally coordinated Si and Al, containing other transition metals incorporated isomorphically or impregnated, such as B, Ga, Fe, Cr, Ge, Ti, V, Mn, Co, Zn, Be, and Cu, are generically referred to as zeotypes.^30^ In this context, the use of waste containing atoms other than silicon and aluminum for the synthesis of zeolites can produce zeotypes, as long as other metals are observed in the crystal lattice along with Si and Al. In addition, whether it is conventional or alternative, such as zeotypes, its application in an adsorption system requires a thorough understanding of the mechanisms of adsorption equilibrium, kinetics, and thermodynamics. This knowledge allows for a comprehension of the interactions that occur between adsorbents and adsorbates through a Lagrangian (micro) approach and to scale the system using an Eulerian (macro) approach.^31,32^

Although there are extensive studies on adsorption, including analyses of adsorption energy, external mass transfer, and adsorption mechanisms, the literature predominantly relies on the Langmuir and Freundlich isotherms, as well as the pseudo-first-order (PFO) and pseudo-second-order (PSO) kinetic models. Frequently, in-depth discussions of alternative models and their derived parameters are limited, which can hinder practical applications or industrial scale-up.

Given the need for effective treatment of effluents containing contaminants like Methylene Blue (MB), this study addresses the residual generation of diatomaceous earth (DE) from beer filtration and clarification processes. It explores the utilization of this residual DE as a silicon source to synthesize zeotypes. We evaluated the adsorption of MB onto these zeotypes, assessing the kinetic, equilibrium, and thermodynamic mechanisms to provide a comprehensive understanding of the interactions between the adsorbent and adsorbate.

## 2 Materials and Methods

### 2.1 Synthesis and Characterization of the Zeotypes

For the synthesis of the zeotypes, the hydrothermal method was applied (Mintova et al.). A mass of 9.29 g of sodium hydroxide (NaOH, P.A. Dinâmica, purity 98%) and 2.05 g of aluminum hydroxide (Al­(OH)_3_, Dinâmica, purity 76.5%) were dissolved in 32 mL of deionized water and magnetically stirred for 10 min. Subsequently, 6.96 g of diatomaceous earth (DE) (SiO_2_ 91.55%; Al_2_O_3_ 1.84%; Na_2_O 1.72%; CaO 1.42%; Fe_2_O_3_ 1.15%; P_2_O_5_ 1.09%; K_2_O 0.88%; and MgO 0.35%) that had been previously dried was added to the mixture and aged at 25 °C under stirring for 24 h, generically referred to as solution 1.

Moreover, 38.65 g of NaOH and 21.21 g of Al­(OH)_3_ were dissolved in 340 mL of deionized water and vigorously mixed in a paddle mixer until completely dissolved (solution 2). The final solution was formed by slowly adding 27.22 g of solution 1 to solution 2, which was also aged for 24 h under similar conditions as solution 1. Finally, the final solution was placed in a hydrothermal reactor and crystallized for 48 h at 100 °C in an oven.

The solids were recovered by vacuum filtration, washed to a pH close to 7, and dried at 110 °C for 24 h. The zeotype obtained in the sodium form (NaZ) underwent an ion exchange procedure with ammonium chloride (NH_4_Cl, P.A. Dinâmica, purity 99.5%) at 1.0 molar in the ratio of 1 g of NaZ/50 mL of NH_4_Cl at 60 °C, forming the zeotype in the ammoniacal form (NH_4_Z). This was calcined at 550 °C for 6 h to obtain the zeotype in the protonic form (HZ).

The crystalline phases present in the diatomaceous earth (DE) and in the zeotypes NaZ and HZ were obtained by X-ray diffraction (XRD) using a Rigaku Mini-Flex 600 diffractometer, with a CuKα radiation source (λ = 0.1542 nm), nickel filter, 15 mA current, and 40 kV voltage. The XRD data were collected in the 2θ range of 5 to 50°, with a step of 0.02° (2θ) and a scanning speed of 10° (2θ)/min.

The concentration of acid sites and the nature of Brønsted (B) and Lewis (L) acid sites were determined by in situ FTIR using adsorbed pyridine as a probe molecule. A transmission cell with calcium fluoride (CaF_2_) windows was used in a Bruker Vertex 70 equipped with a mercury cadmium telluride (MCT) detector. Initially, self-supported pellets of the samples, weighing 10 mg, were made in a press with a force of 3 tons. The pellets were placed in the cell and pretreated at 350 °C under an argon flow (100 mL/min) for 1 h, then cooled to 150 °C. The spectra were collected in the range of 4000 to 625 cm^–1^ with 64 scans and a resolution of 4 cm^–1^. A spectrum was collected at 150 °C as a baseline, and then the samples were saturated in situ with pyridine vapor (2 mL every 2 min). Excess and physically adsorbed pyridine were purged with argon flow at 100 mL/min. Next, the spectrum with chemically adsorbed pyridine was collected and subtracted from the baseline without pyridine. The nature of the acid sites was determined by the bands at 1544 cm^–1^ as Brønsted acid sites and at 1447 cm^–1^ as Lewis acid sites. The concentration of Brønsted and Lewis acid sites in mmol/g was determined according to eqs 1 and 2, respectively, where the absorption coefficients used were εB (1.23 cm μmol^–1^) and εL (1.73 cm μmol^–1^).^33^

1 $$C_{B}\left(\mathrm{mmol}.g^{-1}\right)=\frac{A_{B}\pi r^{2}}{\varepsilon_{B\;}m_{\mathrm{cat}}}$$

2 $$C_{L}\left(\mathrm{mmol}.g^{-1}\right)=\frac{A_{L}\pi r^{2}}{\varepsilon_{L}m_{\mathrm{cat}}}$$

Where:

A_B_: Integrated peak Area in 1544 cm^–1^.

A_L_: Integrated peak Area in 1447 cm^–1^.

r:^2^ Pastille radius in cm.

m_cat_: Pastille weight in mg.

### 2.2 Adsorption Kinects

Kinetic tests were conducted in batch mode with the aim of evaluating the adsorption rate of zeotypes in sodium form (NaZ) and protonic form (HZ) and the DE in the removal of MB present in the liquid phase. The dye solutions of MB had their pH adjusted to the value determined by pHpcz for each of the materials (6.2, 5.2, and 4.8 for NaZ, HZ, and DE, respectively)),^34^ using a NaCl solution (0.1 M) at a ratio of 1 mL of solution to 1 mg of solid. The pH of the NaCl solution was adjusted between 3 and 12 using NaOH and HCl solutions (0.1 mol. L^–^;^1^ the mixture was stirred for 1 h at 28 °C. Subsequently, the solids were recovered by filtration, and the final pH was measured. The pHpcz was determined as the maximum value at which the pH did not vary.

The tests were performed with 25 mL of aqueous dye solution at concentrations of 50, 80, and 110 mg·L^–^ ^1^ and 25 mg of the adsorbent previously dried in an oven at 80 °C for 30 min and cooled in a desiccator. After the start of the process, with agitation in a SOLAB brand Shaker SL 222 at 200 rpm and 28 °C, aliquots were taken over time, which were centrifuged at 2000 rpm for 5 min. At the end, the absorbance of the supernatant was measured using a UV–vis spectrophotometer from Drawell, model Du-8800d, at a wavelength of 664 nm. Using the obtained data, the adsorption capacity (*q*) of the dye achieved by the adsorbents (mg­(MB)/g­(adsorbent)) was determined through eq 3), where *V* is the volume of the aqueous solution (L), *C*_0_ is the initial concentration of the dye in the solution (mg·L^–^,^1^ Ct is the concentration of the dye in the solution at specified times (mg·L^–^,^1^ and m is the Weight of the adsorbent (g). Finally, the kinetic curves were plotted with the amount adsorbed over time (mg.g^–^ ^1^ versus time in minutes.

3 $$q=\frac{V\left(C_{o}-C_{t}\right)}{W}$$

The experimental adsorption data were fitted to the nonlinear kinetic models of Pseudo-First Order (PFO), Pseudo-Second Order (PSO), Elovich, and Avrami, as presented in Table 1. Additionally, the mechanistic behavior of the process was investigated through the regression of the experimental data to the multlinear model of Weber and Morris.

**Table 1:** List of Adsorption Kinetic Models in Integrated Form

Kinetic models	Nonlinear form	Reference
*Pseudo-first Order*	$q_{t}=q_{e}\left(1-e^{-k_{1}t}\right)$	^35^
*Pseudo-second Order*	$q_{t}=\frac{k_{2}q_{e}^{2}t}{1+q_{e}k_{2}t}$	^36^
*Elovich*	$q_{t}=\frac{1}{\beta }\ln\left(1+\alpha \beta t\right)$	^37^
*Avrami*	$q_{t}=q_{e}\left(1-e^{-k_{AV}{\left(t\right)}^{n_{AV}}}\right)$	^32^
*Weber and Morris*	$q_{t}=k_{d}\sqrt{t}+B$	^38^

### 2.3 Adsorption Equilibria

The adsorption equilibrium was studied with initial concentrations of MB solutions ranging from 10 to 140 mg·L^–1^, with an equilibrium time of 20 min. From the obtained data, the adsorption capacity at equilibrium was calculated using eq 1. Using the adsorption capacity values and equilibrium concentration, isotherm curves (q_e_ versus C_e_) were plotted, which were fitted to the nonlinear isotherm models of Langmuir, Freundlich, Redlich-Peterson, Sips, Khan, and Temkin, presented in Table 2. The statistical parameters applied for selecting the model that best fitted the data were the adjusted linear coefficient of determination R^2^adj and the chi-square test (χ^2^).^39^

**Table 2:** List of Adsorption Isotherm Models in Integrated Form

Isotherm	Nonlinear form	Parameters	Reference
*Langmuir*	$q_{e}=\frac{Q_{0}bC_{e}}{1+bC_{e}}$	*Q*_0_,*b*	^40^
*Freundlich*	$q_{e}=K_{F}{C_{e}}^{1/n}$	*K*_*F*_,*n*	^41^
*Temkin*	$q_{e}=\frac{RT}{b_{T}}\ln\left(A_{T}C_{e}\right)$	*A*_*T*_,*b*_*T*_	^42^
*Redlich-Peterson*	$q_{e}=\frac{K_{R}C_{e}}{1+a_{R}C_{e}^{g}}$	*K*_*R*_,*a*_*R*_,*g*	^43^
*Sips*	$q_{e}=\frac{K_{S}{C_{e}}^{\beta_{S}}}{1+a_{S}C_{e}^{\beta_{S}}}$	*K*_*S*_,*a*_*S*_,*β*_*S*_	^44^
*Khan*	$q_{e}=\frac{q_{s}b_{K}C_{e}}{{\left(1+b_{K}C_{e}\right)}^{a_{K}}}$	*q*_*S*_,*b*_*K*_,*a*_*K*_	^45^

### 2.4 Adsorption Thermodynamics

To determine the thermodynamic adsorption parameters of MB on NaZ, HZ, and DE, the equilibrium constant (*K*_ad_) was initially obtained by taking the ratio between the adsorption capacity and the equilibrium concentration (eq 4), multiplying by the mass of the adsorbent used in grams and dividing by the volume of the solution in liters, in order to obtain the dimensionless *K*_ad_. Using the *K*_ad_ values, a graph of ln *K*_ad_ versus 1/T (*T* = 301, 313, and 323 K) was plotted to obtain the equation of the line for interpreting the thermodynamic parameters. From the equation of the line, the slope corresponded to ΔH/R and the intercept corresponded to ΔS/R from eq 4, where R is the universal gas constant (8.314 J·mol^–^.^1^ K^–^.^1^

4 $$K_{\mathrm{ad}}=\frac{q_{e}}{C_{e}}$$

Finally, ΔG was determined by eq 7, which is obtained by equating eqs 5 and 6.

5 $$\Delta G_{\mathrm{Ads}}=\Delta H_{\mathrm{Ads}}-T\Delta S_{\mathrm{Ads}}$$

6 $$\Delta G_{\mathrm{Ads}}=-RT\ln K_{\mathrm{ad}}$$

7 $$\ln K_{\mathrm{ad}}=\frac{\Delta S}{R}-\frac{\Delta H}{RT}$$

## 3 Results and Discussion

### 3.1 Characterization

#### 3.1.1 X-ray Diffraction (XRD)

Figure 1 shows the diffraction patterns of the precursor material (DE), the synthesized materials, and the crystalline phases. It can be observed that, although the DE presents cristobalite (International Diffraction Data Center (ICDD) sheet 01–082–1233) as a crystalline phase (°2 theta = 21.8), the synthesis of the zeotypes was achieved only with the presence of muscovite (ICDD 01–080–0743), without the initial phase of DE. Typically, the use of alternative materials as a silicon source in zeotype synthesis requires structural amorphization through acid pretreatments, which is neither economically nor environmentally friendly.^46^

**Figure 1:**
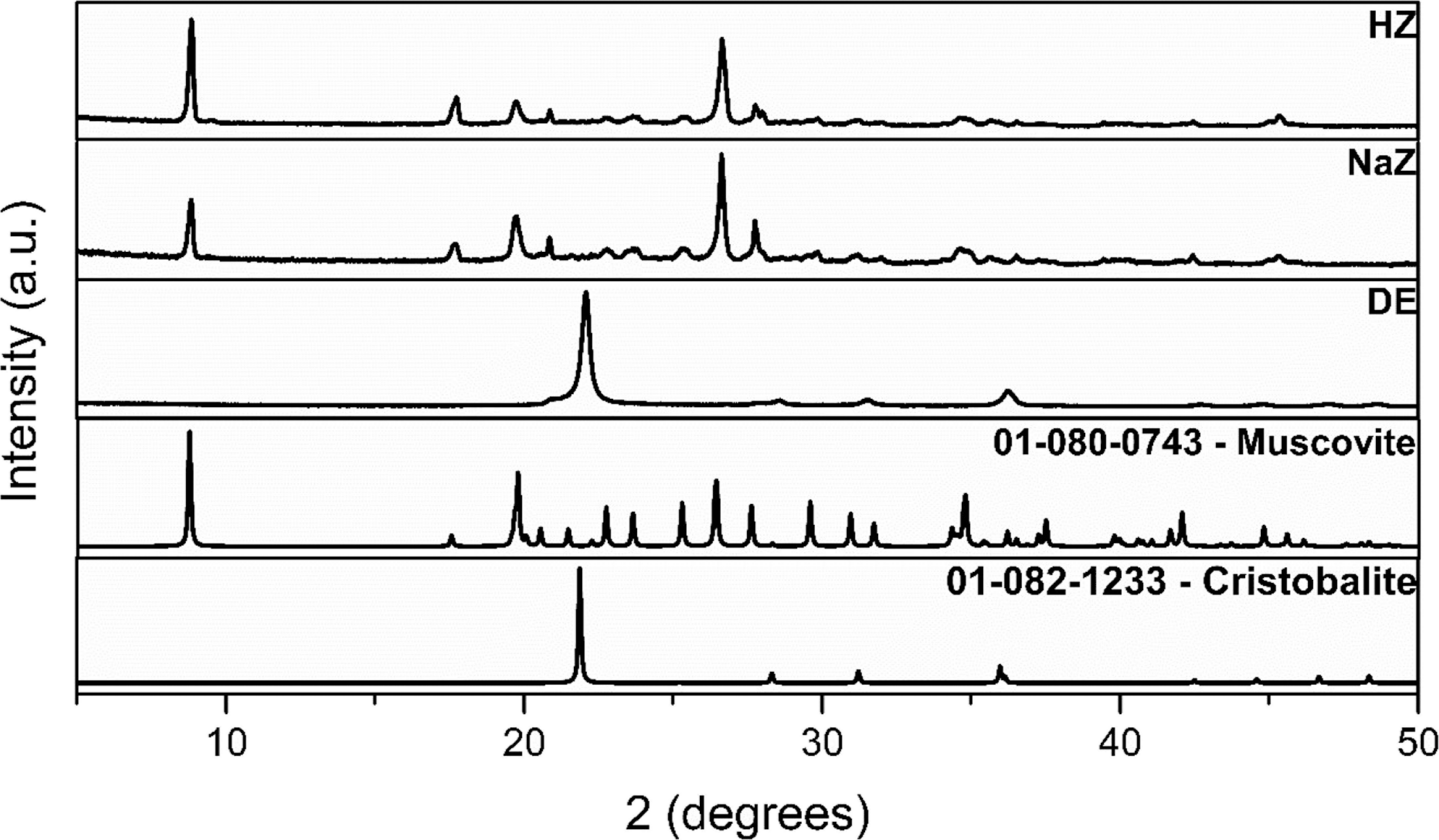
Difratograms of DE, NaZ, HZ, and crystallographic phases.

The use of alternative sources of amorphous silicon promotes the nucleation and orderly growth of tetrahedrally coordinated crystals. Through the similarity in the diffractograms of NaZ and HZ, it can be concluded that the ion exchange procedure was successful, as it did not cause significant changes in the crystalline structure.

#### 3.1.2 Adsorbed Pyridine FTIR

Figure 2 shows the *in situ* FTIR spectra with adsorbed pyridine. It can be observed that the spectrum for DE did not show intensity in the regions attributed to the acidity of B (1544 cm^–1^) and L (1447 cm^–1^). The zeotype NaZ exhibits a slight increase in intensities, while the zeotype in the protonic form (HZ) showed higher intensities. The region at 1544 cm^–1^ is attributed to the bond of H with N of pyridine, forming the pyridinium ion, while the band at 1447 cm^–1^ is attributed to the donation of electron pairs from pyridine to the acid sites of L in the zeotype, which may be linked to structural defects.^33^

**Figure 2:**
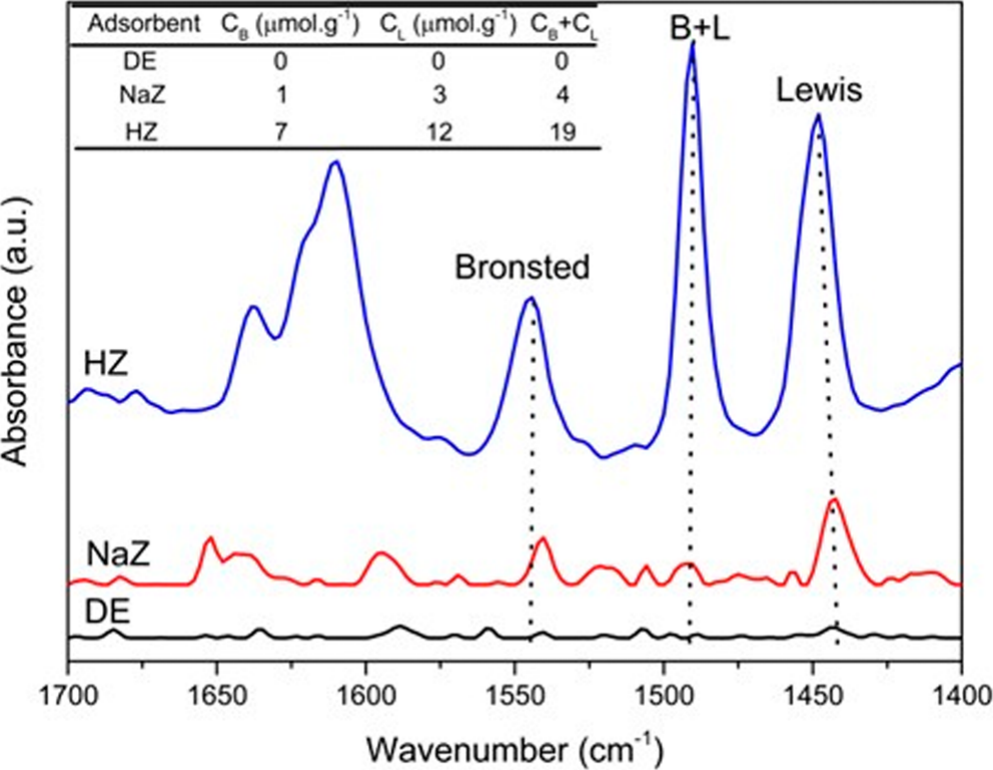
FTIR spectra after in situ Py adsorption at 150 °C collected for DE, NaZ and HZ.

The HZ exhibited greater acidity compared to the other materials, which was expected, as it is present in the protonic form. The quantification of the sites revealed higher L acidity for the zeotypes NaZ and HZ, which can be attributed to structural defects occurring during synthesis.^47^ The presence of acid sites in adsorbent materials is required due to the potential interactions with pollutant adsorbates such as the MB dye.

### 3.2 Adsorption Kinects

Studying adsorption kinetics using empirical models provides valuable insights into the reactions and interactions between the adsorbate and the adsorbent. In this context, the mass transfer from the fluid phase to the solid phase during the adsorption process can be understood through three key steps: external diffusion, intraparticle diffusion, and the attachment of the adsorbate to the adsorption sites.^32^

To enhance the understanding of diffusion and reaction phenomena, empirical models have been developed to accurately depict the adsorption mechanisms. The kinetic models of Pseudo-First Order (PFO), Pseudo-Second Order (PSO), Elovich, and Avrami are valuable for assessing the adsorption rates of species onto the surface of the adsorbent. Conversely, the Weber and Morris kinetic model offers a more comprehensive description of the primary stages involved in the overall process, clarified through the validation of a multilinear system.^32^

Figure 3 illustrates the kinetic curves for the removal of MB using the NaZ zeotype. The experimental data for MB removal at initial concentrations of 50 mg·L^–1^ (Figure 3A), 80 mg·L^–1^ (Figure 3B), and 110 mg·L^–^ ^1^ (Figure 3C) were fitted to the nonlinear kinetic models of Pseudo-First Order (PFO), Pseudo-Second Order (PSO), Elovich, and Avrami. Figure 3D presents the fitting of the experimental data to the Weber and Morris model for MB removal at a concentration of 50 mg·L^–1^.

**Figure 3:**
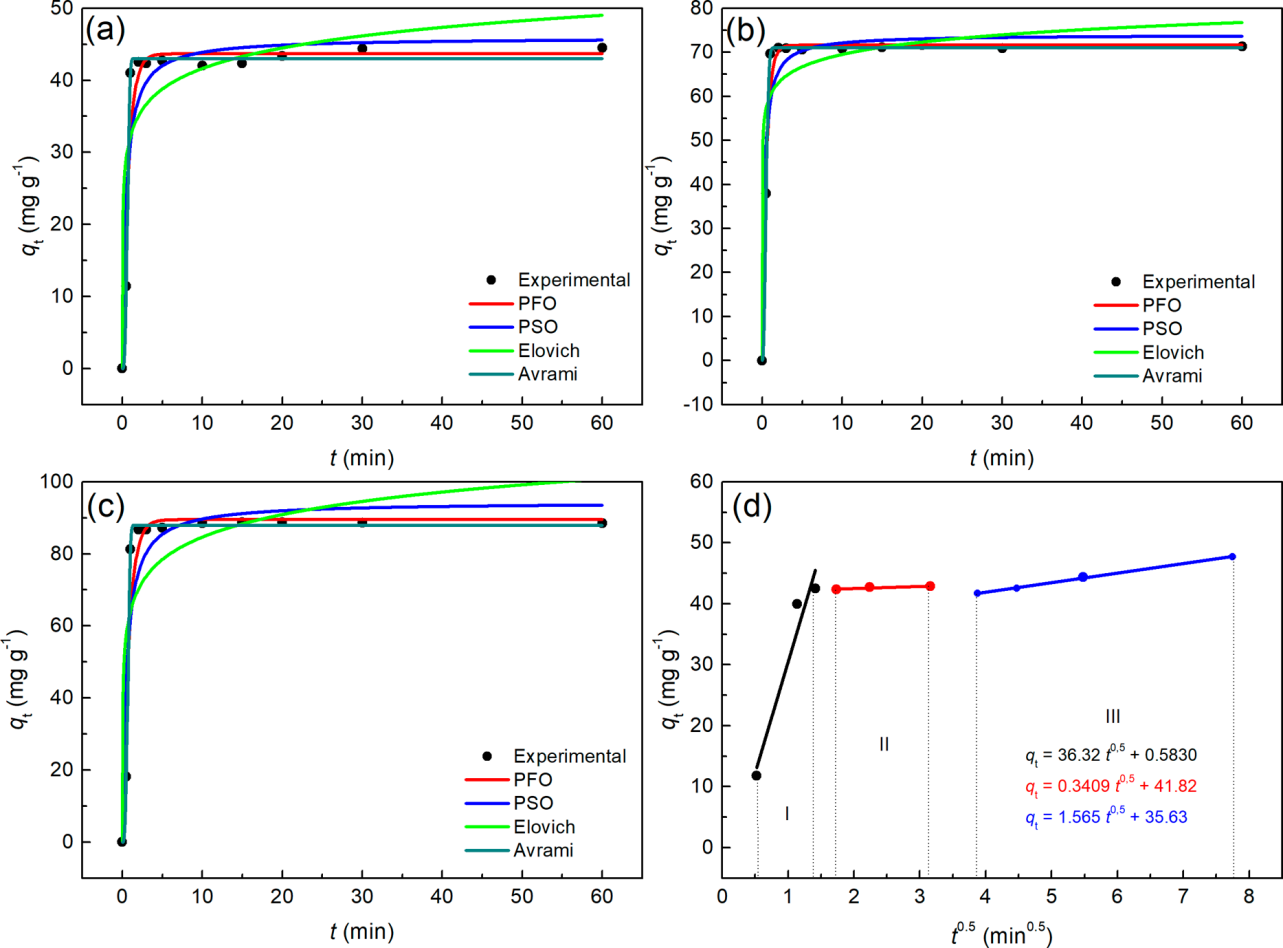
Adsorption kinetic curves obtained for NaZ with adjustment to the PFO, PSO, Elovich and Avrami models at MB concentrations of (a) 50 mg·L^–1^, (b) 80 mg·L^–1^, (c) 110 mg·L^–1^ and (d) adjustment of the Weber and Morris Model at 50 mg·L^–1^. pHzpc (6.2; 5.2 and 4.8), *T* = 28 °C.

From Figure 3, it can be observed that the adsorption equilibrium for MB using NaZ was relatively rapid, achieving equilibrium in less than 5 min across all three concentrations studied. Regarding the fitting of the experimental data, only the Elovich model appears to be less suited compared to the other models. The intraparticle diffusion model of Weber and Morris was applied to evaluate the influence of diffusional phenomena on the MB adsorption process.

The Weber-Morris model describes a multilinear adsorption profile consisting of three distinct stages, each represented by a linear equation, as shown in Figure 3. These stages provide valuable insights into the underlying mass transfer phenomena. According to the model: (i) the first stage corresponds to the diffusion of the adsorbate onto the adsorbent surface; (ii) the second stage is primarily associated with intraparticle diffusion; and (iii) the third stage represents the system reaching equilibrium or a steady state^48^

Adsorption processes where C ≠ 0 indicate the dominance of diffusion resistance in the film, as higher C values correspond to a greater influence of the boundary layer on the overall process rate. Under these conditions, external diffusion becomes the limiting step in adsorption. Conversely, when the constant C is zero (resulting in a linear profile that intersects the origin), intraparticle diffusion governs the mass transfer process, causing the adsorption capacity to vary proportionally to the square root of time^49–51^

The fitting of the experimental data to this model demonstrated that the adsorption process occurred in three stages, with the third stage responsible for the dynamic equilibrium of the process, which is the point at which the rates of adsorption and desorption became equal. This also confirms that the adsorption rate of MB changed between the stages, with times of 1.5 ± 0.5 min (1.22 min) and 3.5 ± 0.5 min (1.87 min), which are relatively short and align with the equilibrium times shown in Figure 3A)–C)-. In the studies by Bazzarella et al., which aimed to evaluate the performance of Cu^2+^-bentonite clay as an alternative adsorbent for the removal of ethylenethiourea from aqueous solution, the transition between stages I and II occurred at 22 min, and from II to III at 1 h and 45 min, by which time the process had reached equilibrium.^51^ Furthermore, each stage is associated with a mechanism that controls the diffusion rate of MB.

Based on the nonlinear fittings shown in Figure 3, the kinetic parameters for the adsorption of MB using NaZ were obtained and are presented in Table 3 for the PFO, PSO, Elovich, and Avrami models, and in Table 4 for the Weber and Morris model. The nonlinear regression data, assessed using the adjusted coefficient of determination (R^2^_adj_) and the chi-squared (χ^2^) values (Table 3), demonstrated good fits for all studied models. However, the best fit was achieved with the Avrami model, which exhibited the highest R^2^_adj_ and the lowest χ^2^ across the three evaluated concentrations.

**Table 3:** Kinetic Parameters Calculated through Nonlinear Regression Treatments of NaZ Experimental Data to PFO, PSO, Elovich, and Avrami

Models\Concentration	50mg·L^–1^	80mg·L^–1^	110mg·L^–1^
*q*_experimental_ (mg g^–1^)	44.48	71.29	88.51
**Pseudo-first Order**			
*k*_*1*_ (min^–1^)	1.337	1.9333	1.218
*q*_*e*_ (mg g^–1^)	43.69	71.63	89.53
*R* ^*2*^_*adj*_	0.9116	0.9750	0.9043
*χ* ^*2*^	20.52	12.83	97.69
**Pseudo-second Order**			
*k*_*2*_ (g mg^–1^ min^–1^)	0.0465	0.0520	0.0201
*q*_*e*_ (mg g^–1^)	45.93	74.01	94.38
*R* ^*2*^_*adj*_	0.8598	0.9368	0.8476
*χ* ^*2*^	32.56	32.41	155.52
**Elovich**			
α (mg g^–1^ min^–1^)	1.004 × 10^4^	1.61 × 10^7^	1.081 × 10^4^
β (mg g^–1^)	0.2425	0.2469	0.1109
*R* ^*2*^_*adj*_	0.7479	0.8638	0.7183
*χ* ^*2*^	58.56	69.91	287.5
**Avrami**			
*k*_*AV*_ (min^–1^)	3.068	3.902	2.564
*q*_*e*_ (mg g^–1^)	43.01	71.03	87.98
*n*_*AV*_	3.309	2.356	3.473
*R* ^*2*^_*adj*_	0.9966	0.9998	0.9992
*χ* ^*2*^	0.7847	0.0907	0.7311

**Table 4:** Kinetic Parameters Calculated by Fitting the Experimental Data of MB Adsorption at 50 mg·L^–^ ^1^ Using the NaZ to the Weber and Morris Model

Weber and Morris	Stage I	Stage II	Stage III
*K*_*d*_ (mg g^–1^min^–0.5^)	36.32	0.3409	1.565
*C* (mg g^–1^)	0.5830	41.82	35,63

According to the kinetic parameters obtained from the Weber and Morris model (Table 4), the parameter *C* provides insight into the thickness of the mass transfer boundary layer; that is, a higher value of *C* indicates a greater influence of this layer. In this case, a *C* value of 0 signifies that the kinetics of the process were controlled by intraparticle diffusion. Conversely, for processes where *C* ≠ 0, there will be a contribution from film diffusion resistance. Since the values of *C* presented in Table 4 are greater than zero, it can be concluded that the mass transfer processes via intraparticle diffusion were affected by the boundary layer. Thus, the adsorption of MB on the NaZ zeotype was influenced by film diffusion, and due to the multiple stages, intraparticle diffusion also played a role. Based on these results, the Avrami model is indicated as the one that best describes the adsorption data of MB on NaZ, as it aligns with the existence of more than one rate-limiting step and a kinetic process with variable speed throughout. The literature also reports the fitting of kinetic data to the Avrami model, which is further described by Weber and Morris.^51^

The values of *k*_AV_ and *k*_2_ correspond to the Avrami and Pseudo-Second Order (PSO) models, respectively. Both models are useful for describing kinetic mechanisms; however, the constant *k*_AV_ provides a more accurate assessment of the kinetic mechanism compared to the constant *k*_2_. This is because the Avrami constant is independent of the initial concentration of the adsorbate, with units of min^–1^, whereas the PSO constant is strongly dependent on the initial concentration of the adsorbate in the solution, with units of g.mg^–1^.min^–1^.^14^

Figure 4 displays the kinetic adsorption curves of Methylene Blue (MB) using the protonic zeotype (HZ), with nonlinear fittings for the same models and parameters studied for NaZ. It is evident that the adsorption equilibrium time for MB with the HZ zeotype was relatively longer, approximately 20 min for the 50 mg·L^–1^ and 110 mg·L^–1^ solutions, and around 5 min for the 80 mg·L^–1^ solution, indicating a different behavior compared to NaZ. The data fitted to the Weber and Morris model reveal that the adsorption process also occurred in three stages. By comparing Figures 3 and 4, kinetic equilibrium was reached at lower adsorption capacities (*q*_t_) for HZ across all analyzed concentration.

**Figure 4:**
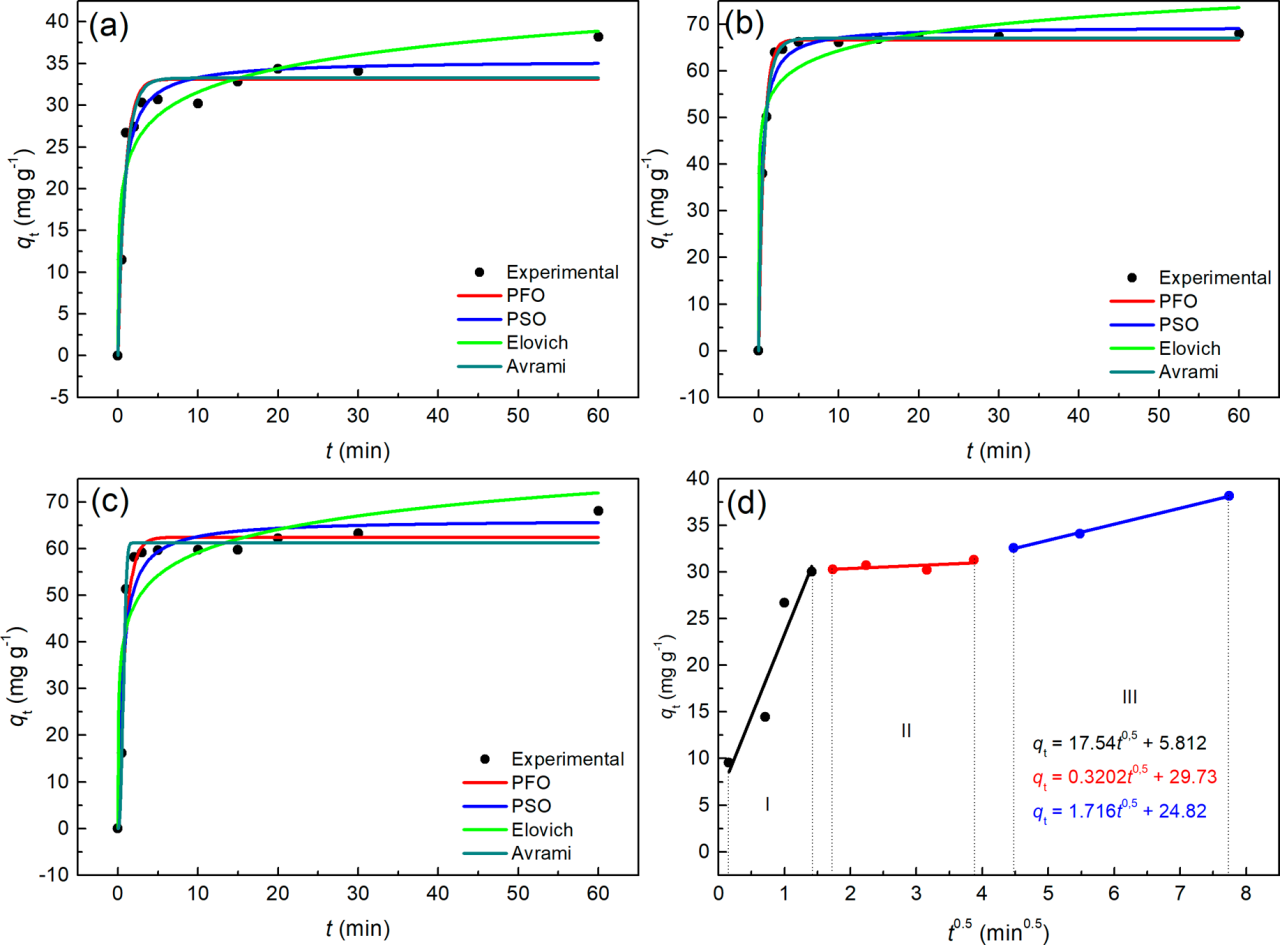
Adsorption kinetic curves obtained for HZ with adjustment to the PFO, PSO, Elovich and Avrami models at MB concentrations of (a) 50 mg·L^–1^, (b) 80 mg·L^–1^, (c) 110 mg·L^–1^ and (d) adjustment of the Weber and Morris Model at 50 mg·L^–1^. pHzpc (6.2; 5.2 and 4.8), *T* = 28 °C.

Table 5 presents the parameters calculated through the nonlinear fitting of the Pseudo-First Order (PFO), Pseudo-Second Order (PSO), Elovich, and Avrami models to the experimental data of MB adsorption using HZ, while Table 6 displays the parameters of the Weber and Morris model for MB adsorption data at 50 mg·L^–1^. According to the overlap of the curves in Figure 5, along with the *R* ^*2*^_*adj*_ and χ^2^ values in Table 5, all models accurately describe the observed experimental behavior. However, among the models examined, the Avrami model exhibited the best regression indicators. This suggests a strong agreement with the Weber and Morris model, which outlines the adsorption kinetic mechanism in three stages, similar to the results obtained for NaZ. The fitting to the Weber and Morris model reveals a C≠0 (Table 6), supporting the understanding that there was a kinetic contribution from film diffusion, alongside the influence of the mass transfer boundary layer.

**Table 5:** Kinetic Parameters Calculated through Nonlinear Regression Treatments of the HZ Experimental Data to the PFO, PSO, Elovich, and Avrami Model

Models\Concentration	50mg·L^–1^	80mg·L^–1^	110mg·L^–1^
*q*_experimental_ (mg g^–1^)	38.16	67.93	68.04
**Pseudo-first Order**			
*k*_*1*_ (min^–1^)	1.114	1.544	1.139
*q*_*e*_ (mg g^–1^)	33.13	66.68	62.46
*R* ^*2*^_*adj*_	0.9332	0.9962	0.9394
*χ* ^*2*^	8.463	1.642	28.59
**Pseudo-second Order**			
*k*_*2*_ (g mg^–1^ min^–1^)	0.0457	0.0406	0.0258
*q*_*e*_ (mg g^–1^)	35.38	69.47	66.25
*R* ^*2*^_*adj*_	0.9428	0.9878	0.9067
*χ* ^*2*^	7.251	5.2827	44.02
**Elovich**			
α (mg g^–1^ min^–1^)	9.463 × 10^2^	1.34 × 10^5^	2.83 × 10^3^
β (mg g^–1^)	0.2453	0.1938	0.1400
*R* ^*2*^_*adj*_	0.9018	0.9229	0.8133
*χ* ^*2*^	12.45	33.57	88.14
**Avrami**			
*k*_*AV*_ (min^–1^)	1.088	1.466	1.819
*q*_*e*_ (mg g^–1^)	33.27	67.02	61.24
*n*_*AV*_	0.9320	0.8430	2.565
*R* ^*2*^_*adj*_	0.9589	0.9979	0.9809
*χ* ^*2*^	9.502	1.041	9.000

**Table 6:** Kinetic Parameters Calculated by Fitting the Experimental Data of MB Adsorption at 50 mg·L^–1^ Using the HZ to the Weber and Morris Model

Weber and Morris	Stage I	Stage II	Stage III
*K*_*d*_(mg g^–1^min^–0.5^)	17.54	0.3202	1.716
*C* (mg g^–^ ^1^	5.812	29.73	24.82

**Figure 5:**
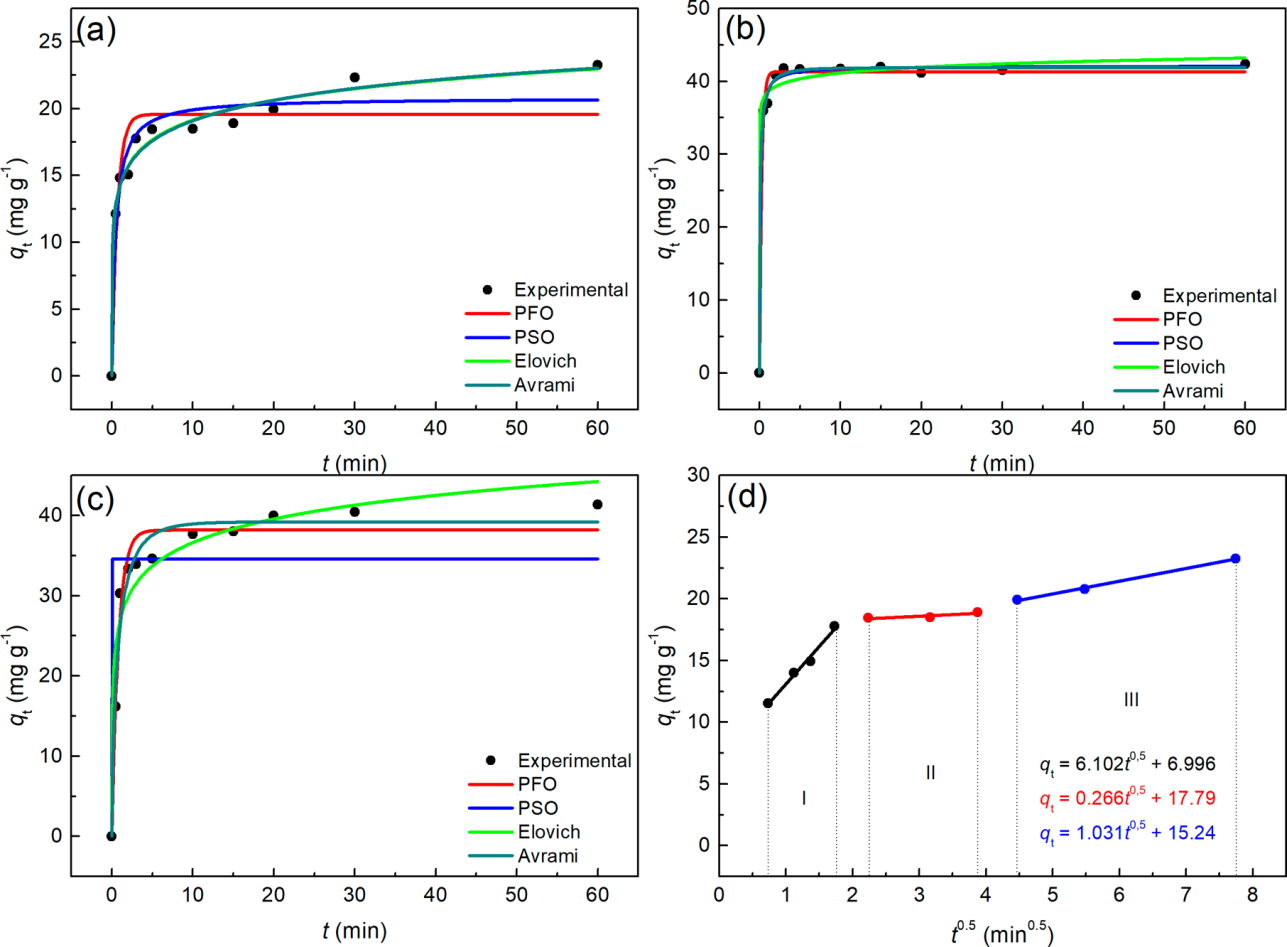
Adsorption kinetic curves obtained for DE with adjustment to the PFO, PSO, Elovich and Avrami models at MB concentrations of (a) 50 mg·L^–1^, (b) 80 mg·L^–1^, (c) 110 mg·L^–1^ and (d) adjustment of the Weber and Morris Model at 50 mg·L^–1^. pHzpc (6.2; 5.2 and 4.8), *T* = 28 °C.

Figure 5 illustrates the kinetic model curves fitted to the adsorption data of Methylene Blue (MB) using Diatomaceous Earth (DE) as the adsorbent, which served as a silica source in the synthesis of the NaZ and HZ zeotypes. A comparison of Figures 3, 4, and 5 reveals that the adsorption capacity of the synthesized zeotypes significantly exceeds that of DE, which was used as the precursor material.

**Figure 6:**
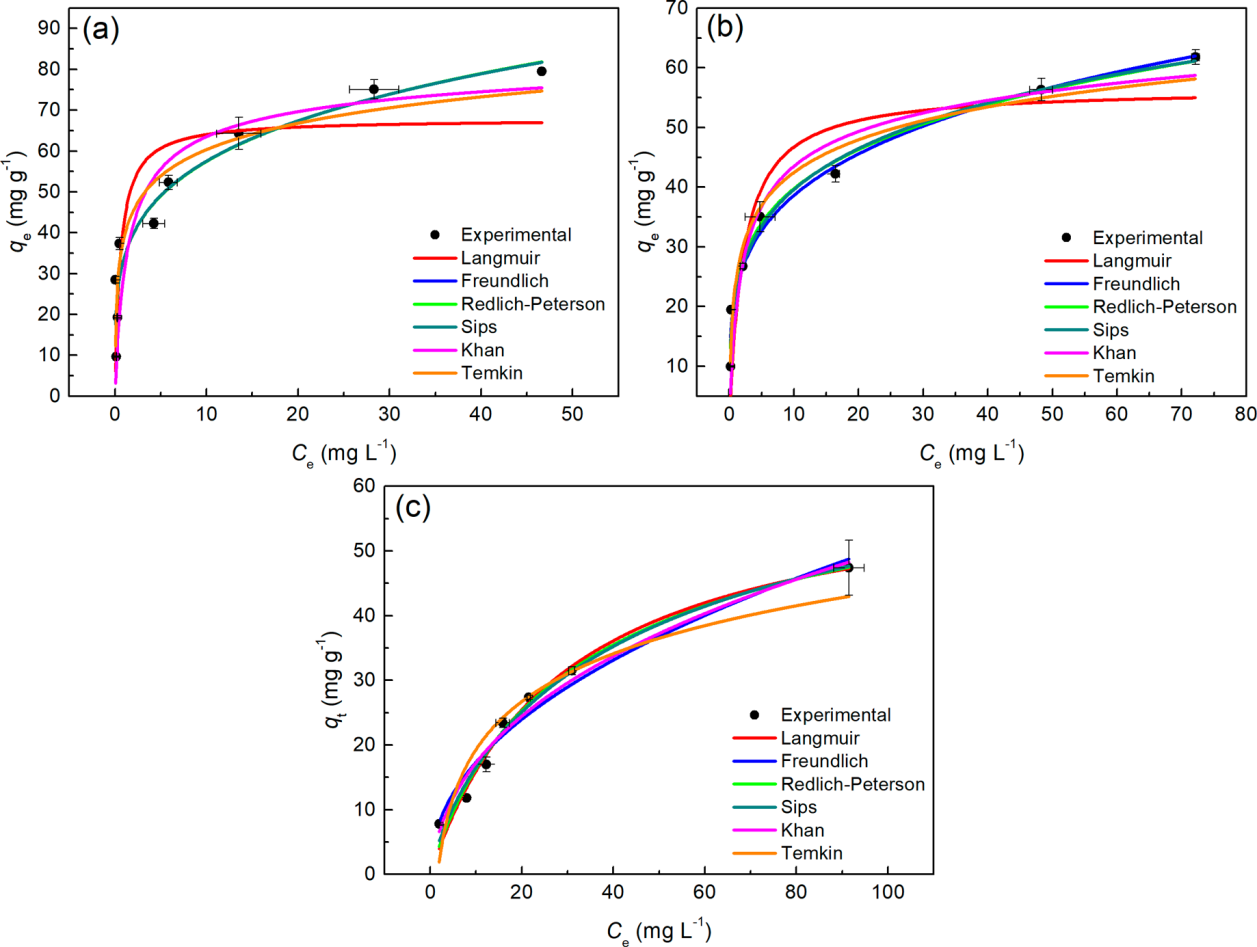
Adsorption isotherms MB fitted to the Langmuir, Freundlich, Redlich-Peterson, Sips, Khan and Temkin adsorption equilibrium models related for: (a) NaZ; (b) HZ and (c) DE. pHzpc (6.2; 5.2 and 4.8), *T* = 28 °C.

The behavior of the curves is similar to those observed for NaZ (Figure 3) and HZ (Figure 4), indicating a rapid adsorption kinetics; however, the kinetic equilibrium was reached in approximately 20 min for the solutions with initial concentrations of 50 mg·L^–^ ^1^ and 110 mg·L^–1^ (Figure 5A,C), which is a longer equilibrium time compared to NaZ and similar to HZ. The stages of the adsorption process are divided into three, as determined by the intraparticle diffusion model (Figure 5D). The kinetic parameters obtained through the nonlinear fitting of the experimental data for MB adsorption using DE for the PFO, PSO, Elovich, and Avrami models are presented in Table 7, while the kinetic parameters for the Weber and Morris model are shown in Table 8. According to the *R* ^*2*^_*adj*_ and χ^2^ values, the experimental data for DE also fit best to the Avrami model.

**Table 7:** Kinetic Parameters Calculated through Nonlinear Regression Treatments of the DE Experimental Data to the PFO, PSO, Elovich, and Avrami Models

Models\Concentration	50mg·L^–1^	80mg·L^–1^	110mg·L^–1^
*q*_experimental_ (mg g^–1^)	23.27	42.43	41.37
**Pseudo-first Order**			
*k*_*1*_ (min^–1^)	1.456	3.727	1.234
*q*_*e*_ (mg g^–1^)	19.58	41.32	38.19
*R* ^*2*^_*adj*_	0.8832	0.9893	0.9601
*χ* ^*2*^	4.704	1.647	6.281
**Pseudo-second Order**			
*k*_*2*_ (g mg^–1^ min^–1^)	0.1050	0.2538	2.760
*q*_*e*_ (mg g^–1^)	20.82	42.10	34.58
*R* ^*2*^_*adj*_	0.9456	0.9963	0.6558
*χ* ^*2*^	2.189	0.5584	54.37
**Elovich**			
α (mg g^–1^ min^–1^)	1.65 × 10^3^	4.086 × 10^4^	2.380 × 10^3^
β (mg g^–1^)	0.4675	0.8703	0.2356
*R* ^*2*^_*adj*_	0.9832	0.9882	0.9289
*χ* ^*2*^	0.6745	1.8090	11.21
**Avrami**			
*k*_*AV*_ (min^–1^)	0.5893	2.479	1.092
*q*_*e*_ (mg g^–1^)	32.00	41.87	39.22
*n*_*AV*_	0.1879	0.4099	0.6581
*R* ^*2*^_*adj*_	0.9899	0.9966	0.9605
*χ* ^*2*^	0.7975	0.5220	6.230

**Table 8:** Kinetic Parameters Calculated by Adjusting the Experimental Data of MB Adsorption at 50 mg·L^–1^ Using DE for the Weber and Morris Model

Weber and Morris	Stage I	Stage II	Stage III
*K*_*d*_(mg g^–1^min^–0.5^)	6.102	0.266	1.031
*C* (mg g^–1^)	6.996	17.79	15.24

A brief analysis of the parameters obtained from the evaluated kinetic models allows for a comparison between the observed values and the theoretical expectations for each model. The model that best describes the kinetic mechanism of MB adsorption onto the three studied materials (NaZ, HZ, and DE) in terms of sorption rate is the Avrami model. This is supported not only by the statistical tests mentioned earlier (*R* ^*2*^_*adj*_ and χ^2^) but also by the inadequate fitting of the parameters from the other models, which do not align with theoretical predictions.

For instance, the constant *k*_*1*_ (min^–1^) in the PFO model is associated with the sorption rate; its inverse (1/*k*_*1*_) represents the time required for the adsorption process to reach equilibrium. The value of this constant is inversely proportional to the initial concentration of the adsorbate. Consequently, as the initial concentration increases, *k*_*1*_ decreases, resulting in a longer time needed to achieve equilibrium,^32,52,53^

Although the basic kinetic concept suggests that the intrinsic adsorption rate should be independent of the initial concentration, the empirical *k*_*1*_ values derived from the PFO model also reflect influences such as diffusion limitations, heterogeneity of adsorption sites, and other complexities of the system.^54^ However, in the present study, k1 values decreased with increasing MB concentration to 110 mg·L^–1^ (Tables 3, 5 and 7). Therefore, the observed behavior indicates that the PFO model, in its current formulation, may not adequately capture the full complexity of the dye adsorption mechanism for the adsorbents analyzed. This observation is consistent with the findings of Vaghetti et al., who reported that *k*_*1*_ did not decrease with increasing initial concentration during the removal of Cu (II), Mn (II), and Pb (II) using a pecan shell biosorbent, ultimately leading to poor fits for the PFO model.

The constant *k*_*2*_ of the PSO model represents the second-order adsorption rate concerning the adsorptive site, meaning that the uptake rate is second-order with respect to the available active sites.^36^ Like *k*_*1*_, the constant *k*_*2*_ represents a time scale that decreases with the increase in initial concentration. In the present study, the values of the constant *k*_*2*_ only decrease with the increase in C_0_ for the adsorption of MB using HZ. However, the literature indicates that the PSO model is applicable to a wide range of kinetic processes in nature.^32^ For instance, Chinoune et al., in their study of the adsorption kinetics of the recalcitrant organic dyes Procion Blue HP and Remazol Brilliant Blue R using modified bentonite, found that the PSO model fit the experimental data well, and that the values of *k*_*2*_ decreased with increasing C_0_.^55^

In the Elovich model, the parameters α and β represent the initial adsorption rate and the desorption constant, respectively. The parameter β is associated with the extent of surface coverage and the activation energy. This model is satisfactorily applied in environments with significant surface heterogeneity.^56^ However, although the three adsorbent materials used for the removal of MB exhibit heterogeneous surface structures from a morphological perspective (Figure S1), the Elovich model does not adequately describe the process. This is because the initial adsorption rate does not decrease with an increase in *C*_*0*_. Furthermore, this initial rate is not slow, which is a condition required for the applicability of this model.

On the other hand, the Avrami kinetic model is of the fractal (or fractional) type, meaning it describes a kinetic system with a rate coefficient that is time dependent. Thus, the kinetic constant *k*_*AV*_ represents the adsorption rate and can take on distinct values, as it is a constant dependent on another constant *n*. The value of *n* can be either integer or fractional, and when *n* = 1, the model corresponds to the PFO model (Table 1).^57^

This model indicates that the adsorption process varies with time due to the contributions from multiple stages of the mechanism to the overall adsorption rate, as reflected in the fractional reaction order (*n*_AV_) present in the model’s equation. This distinguishes it from the other models previously mentioned, as it exhibits a temporal dependence during the adsorption process. The Avrami kinetic model provided the best fit for the experimental adsorption data of MB across the three evaluated adsorbents. Although the adsorption rate *n*_AV_ does not decrease with increasing C_0_, this model still effectively describes the kinetic mechanism with fractional behavior and time-dependent variations due to contributions from multiple stages to the overall adsorption rate, as revealed by the Webber and Morris model.^51^

A brief comparison between the experimentally determined adsorption capacity (*q*_experimental_) and the capacities predicted by the PFO, PSO, and Avrami models (Tables 3, 5, and 7) reveals that the values are closely aligned across the different concentrations and adsorbents studied. In this context, the adsorption capacity serves as a parameter that further supports the overall fitting of the models to the experimental data.

**Figure 7:**
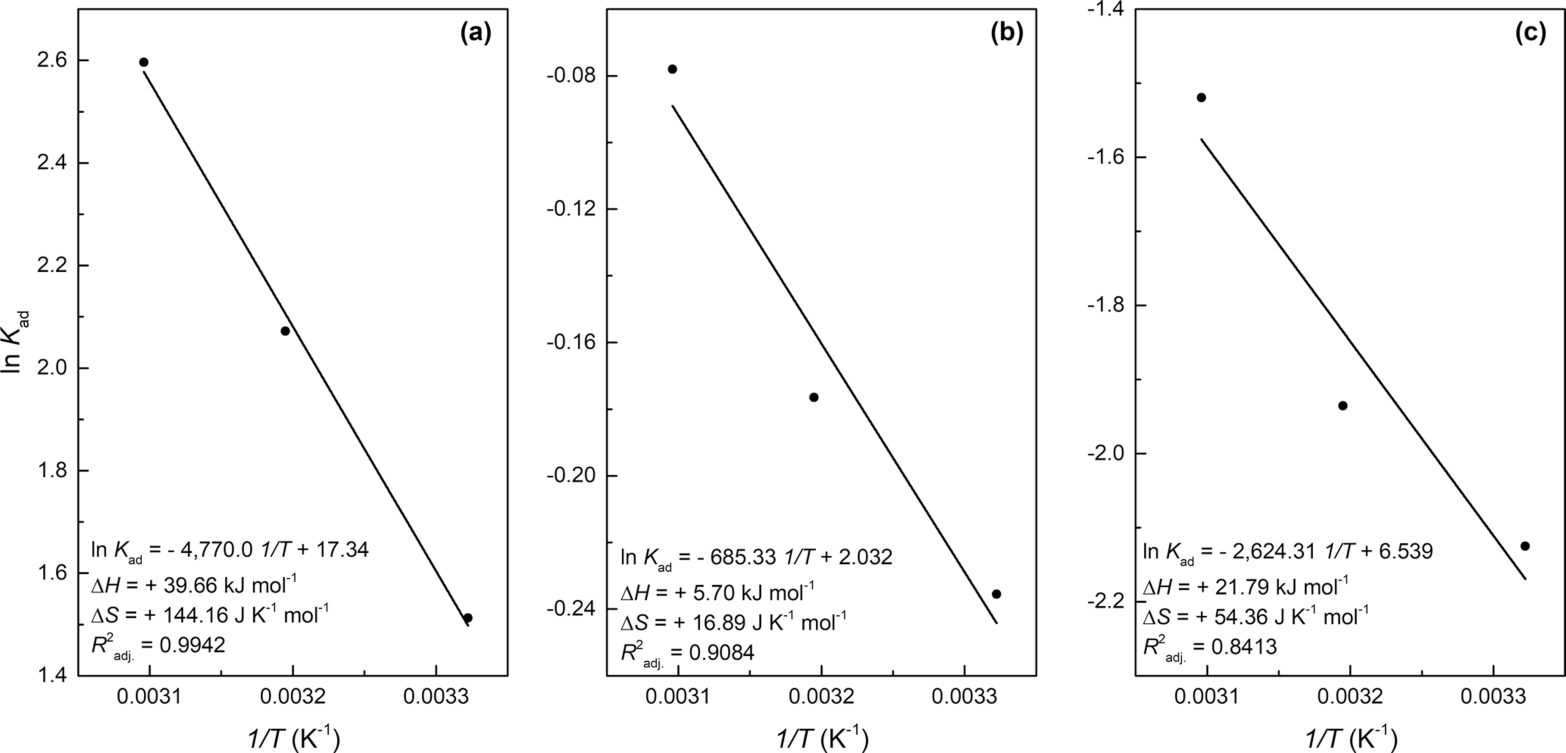
Van’t Hoff plot for the adsorption of MB dye onto the surface of NaZ (a), HZ (b), and DE (c).

### 3.3 Adsorption Equilibria

The study of adsorption equilibrium at constant temperature and pH describes the relationship between the adsorption capacity of an adsorbent (*q*_*e*_) and the concentration of the remaining adsorbate in the solution after equilibrium is reached (*C*_*e*_).^13^ This relationship plays a crucial role in understanding the interactions between the adsorbate and adsorbent through retention or release, as well as the surface properties. Therefore, its understanding is achieved through adsorption isotherms and their parameters.^51^

There is a wide variety of adsorption equilibrium isotherm models. All of these models were formulated based on kinetic approaches (Langmuir 1916) and thermodynamic principles^58^ and the potential theory.^59^ The fundamental approach through kinetics indicates that a dynamic equilibrium state is reached when the rates of adsorption and desorption become equal. From a thermodynamic perspective, there are numerous ways to derive the isotherms, with potential theory being considered the third kinetic approach.^60^ Thus, to describe the interactions between the dye MB and the zeotypes NaZ, HZ, and DE at dynamic equilibrium through a kinetic approach, the experimental data were fitted to several isotherm models: Langmuir, Freundlich, Temkin, Redlich-Peterson, Sips, and Khan. The first three models are classified as two-parameter isotherms, while the latter three are three-parameter isotherms.

Figure 6 illustrates the isotherms and their fits to the dynamic equilibrium adsorption data of MB, with (A) for NaZ, (B) for HZ, and (C) for DE. It is evident that all models follow the trend of the experimental data; however, the Langmuir isotherm shows a clear discrepancy (red curve in Figure 6 (A) and (B)) for the zeotypes NaZ and HZ, respectively. Further insights into this behavior can be gained through the analysis of the physicochemical parameters.

The Langmuir isotherm was originally developed to describe gas–solid adsorption on activated carbon, based on the following assumptions: (1) a fixed quantity of well-defined sites on the adsorbent’s surface, (2) monolayer adsorption, (3) adsorption of a single species per active site, (4) all sites are energetically equivalent, (5) at equilibrium, the rates of adsorption and desorption are equal, and (6) the adsorbate species do not interact with each other.^40^ In contrast, the Freundlich isotherm model is based on empirical foundations that differ from those of Langmuir, including (1) multilayer adsorption, (2) distinct affinities on a heterogeneous surface, and (3) a nonuniform energy distribution.^51,60^

Similar to the Langmuir adsorption isotherm, other isotherms are also essential for elucidating the interactions between textile dyes and alternative adsorbents. These models facilitate a deeper understanding of the mechanisms involved in the adsorption process. The Freundlich isotherm, in particular, posits that adsorption occurs on a heterogeneous surface where interactions among adsorbed molecules take place. This model is particularly applicable to systems where adsorption occurs in multiple layers and involves heterogeneous energy distributions.^41,61^

The Temkin isotherm accounts for the influence of adsorbate–adsorbent interactions by assuming a uniform distribution of binding energies up to a maximum value. This model is particularly useful for describing systems in which the adsorption energy decreases with increasing surface coverage,^42,61,62^ Conversely, the Sips isotherm integrates characteristics from both the Langmuir and Freundlich models, making it applicable to heterogeneous surfaces. It predicts behavior akin to that of the Freundlich model at low concentrations and resembles the Langmuir model at high concentrations.^44^

The Redlich-Peterson model is a hybrid model that integrates the Langmuir and Freundlich models, demonstrating flexibility in describing both homogeneous and heterogeneous systems.^43,63^ Additionally, the Khan model introduces a modification of the Langmuir model by incorporating additional parameters that more accurately characterize complex adsorption systems, such as those encountered in the removal of dyes from aqueous solutions using alternative adsorbents,^45,61^ as is relevant to the present study.

These models not only facilitate the experimental adjustment of adsorption data but also enable the inference of the affinity of adsorbents for dyes and their maximum adsorption capacity. This capability aids in the development of more efficient and sustainable materials for the treatment of textile effluents, while also serving as a valuable tool for process scaling.

Table 9 presents the parameters obtained from the nonlinear fittings of the isotherms displayed in Figure 6 to the experimental data. Based on the values of *R* ^*2*^_*adj*_ and χ^2^ (Table 9), the Freundlich isotherm provided the best fit for the dynamic equilibrium of MB adsorption on the zeotype NaZ. This behavior indicates a heterogeneous type of interaction; the parameter *n*_*F*_ (4.356) suggests that the physical adsorption process is favorable, as it falls between 1 and 10. Additionally, *K*_*F*_ (33.87 (mg.g^–1^/(mg·L^–1^)^n^) indicates a high adsorption capacity of MB by NaZ.

**Table 9:** Parameters Calculated through Nonlinear Regression Treatments of Experimental Data to Langmuir, Freundlich, Redlich-Peterson, Sips, Khan and Temkin Isotherms for MB Adsorption in NaZ, HZ, and DE

Langmuir	NaZ	HZ	DE
*K*_*L*_ (L mg^–1^)	1.693	0.4734	0.0345
*q*_*max*_ (mg g^–1^)	67.835	56.581	62.263
*R* ^*2*^_*adj*_	0.7167	0.8244	0.9746
*χ* ^*2*^	168.89	63.00	4.539
**Freundlich**			
*K*_*F*_ (L mg^–1^)	33.874	22.241	5.906
*n*_*F*_	4.356	4.175	2.139
*R* ^*2*^_*adj*_	0.9000	0.9778	0.9609
*χ* ^*2*^	59.520	7.965	6.9886
**Temkin**			
*aT* (L mol^–1^)	65.333	21.131	0.6066
*bt* (L g^–1^)	2.663 × 10^3^	3.120 × 10^2^	2.315 × 10^2^
*R* ^*2*^_*adj*_	0.8479	0.9602	0.8876
*χ* ^*2*^	90.66	13.636	20.122
**Redlich-Peterson**			
*K*_*RP*_ (L g^– 1^)	4.716 × 10^10^	2.942 × 10^2^	2.475
*a*_*RP*_ (mg L^– 1^)^− β^	1.392 × 10^9^	11.911	0.0645
β	0.7704	0.7874	0.900
*R* ^*2*^_*adj*_	0.8835	0.9787	0.9689
*χ* ^*2*^	69.440	7.6230	5.556
**Sips**			
*K*_*S*_ (L mg^–1^)	0.0123	0.1368	0.0428
*q*_*max*_ (mg g^–1^)	68.563	38.386	74.446
*n*_*S*_	4.280	3.308	1.211
*R* ^*2*^_*adj*_	0.8835	0.9746	0.9270
*χ* ^*2*^	69.435	9.117	5.012
**Khan**			
*K*_*Khan*_ (L mg^–1^)	0.9437	0.9016	0.6251
*q*_*max*_ (mg g^–1^)	62.725	39.86	11.43
*n*_*K*_	1.002	1.000	1.002
*R* ^*2*^_*adj*_	0.6700	0.8706	0.9112
*χ* ^*2*^	196.57	40.401	11.440

The adsorption of MB on other adsorbents, such as Fe_3_O_4_ nanoparticles, exhibited similar behavior to that observed in this study; however, the adsorption capacity (*K*_*F*_) was lower.^3^ In addition to the Langmuir and Freundlich isotherm models, another well-documented isotherm in the literature is the Temkin isotherm, which is described by two adjustable parameters. This model assumes that the heat of adsorption for all adsorbed molecules decreases linearly with coverage, rather than logarithmically at extreme concentrations (both high and low).^64^

For HZ, the Freundlich, Redlich-Peterson, and Sips isotherms demonstrated good fits. However, for the three-parameter models (Redlich-Peterson and Sips), *b* = 0.7874 and n_S_ = 3.308 indicate that the isotherms do not approach Langmuir, as they differ from 1. Therefore, the Freundlich isotherm best represents the interactions between MB and the zeotype HZ. Like NaZ, the physical process is also favorable, albeit with a lower adsorption capacity *K*_*F*_ (22.241 L.mg^–1^). Three-parameter isotherm models, such as those of Redlich-Peterson, Sips, and Khan, represent hybrid isotherms, meaning they are derived from the combination of two distinct models.

For example, the Redlich-Peterson isotherm is derived from the Langmuir and Freundlich isotherms. This versatile model represents dynamic equilibrium across a wide range of concentrations in both homogeneous and heterogeneous systems.^65^ At high concentrations, the Redlich-Peterson isotherm approximates the Freundlich isotherm, while at low concentrations, it approaches the ideal conditions of the Langmuir isotherm.^66^ The Sips isotherm model, like the Redlich-Peterson model, is derived from the combination of the Langmuir and Freundlich isotherms for adsorption in heterogeneous systems, aiming to address the limitation of increasing adsorbate concentration associated with the Freundlich isotherm. Thus, the Sips model converges with the Freundlich isotherm at low concentrations, while at high concentrations, it predicts monolayer adsorption, approaching the Langmuir isotherm.^44,60^

Direct comparison between two- and three-parameter adsorption isotherms involves varying degrees of complexity and can present challenges. Nevertheless, this approach remains prevalent in the literature,^51,67^ and is deemed valid because model adjustments are based on statistical metrics such as R^2^ and χ^2^, which assess the quality of the models’ fit to experimental data, irrespective of the number of parameters involved. Additionally, another aspect that supports and legitimizes the comparison between isotherms with differing numbers of parameters is the physicochemical interpretation; each model is formulated based on distinct hypotheses regarding the adsorption mechanism.

The experimental data for the dynamic equilibrium adsorption of MB using DE showed good fits to the Langmuir, Freundlich, and Redlich-Peterson isotherms. However, since the parameter *b* is different from 1, the Redlich-Peterson isotherm does not reduce to the Langmuir isotherm. Consequently, the favorable adsorption process exhibited a lower adsorption capacity (>*K*_*F*_) for DE compared to the other adsorbents. This behavior was expected, given that DE was used as a silica source in the synthesis of the NaZ and HZ zeotypes.

The results obtained for the adsorption of MB in NaZ, HZ and DE demonstrate that these materials present competitive performance in dye removal in aqueous solution, mainly considering factors such as availability, cost and chemical stability. Although there are adsorbents with significantly higher adsorption capacities in the literature, as shown in Table 10, for information purposes. Some of these materials have limitations, such as complex synthesis, high cost or difficulty in regeneration. For example, zeolitic materials in commercial form, such as MFI, often have high adsorption capacity, but may be economically unviable for large-scale applications.

**Table 10:** Comparative Values of the Maximum Adsorption Capacity of MB on Different Adsorbents

Adsorbent	Q (mg.g^–1^)	C_0_(mg·L^–1^)	References
NaZ	88.51	110	This study
HZ	68.04	110	This study
DE	41.37	110	This study
Nanoparticle of ZrO_2_	23.26	25	^68^
Cellulose Nanocrystals	64.93	100	^12^
Nanoparticle of Fe_2_O_3_	10.47	15	^69^
MgAl-LDH/Biochar	407	100	^63^
Nanosheet MFI zeolite	476	250	^70^
Analcime and cancrinite zeolites	27	100	^71^
NaX	127	140	^72^
Activated carbon	220	250	^73^

Therefore, the choice of adsorbent must consider not only the maximum adsorption capacity, but also the practical feasibility and sustainability of the process. Furthermore, the comparison of the ability of an adsorbent to retain an adsorbate must be made between materials with similar chemical structures.

Several other empirical adsorption isotherm models have been proposed to elucidate the interactions between adsorbate and adsorbent, addressing the limitations encountered by other models, such as the Khan isotherm.^45^ This generalized model is suitable for pure solutions where its maximum adsorption capacity (*q*_*max*_) can be accurately determined, yielding relatively high correlation coefficients and minimal chi-square values.^60^

### 3.4 Adsorption Thermodynamics

Temperature can significantly influence the adsorption process through two distinct effects. First, it reduces the viscosity of the solution, thereby increasing the rate of transfer of adsorbate molecules or ions in the external boundary layer and within the internal pores of the adsorbent particles. Second, it alters the adsorption equilibrium of the adsorbate with respect to a specific adsorbent.^66,74^

Figure 7 describes the Van’t Hoff plot for the adsorbents proposed in the present study. Based on the linearization of the Van’t Hoff equation, it was possible to obtain the thermodynamic parameters presented in Table 11.

**Table 11:** Thermodynamic Parameters Obtained in the Adsorption of MB Dye by NaZ, HZ, and DE between Temperatures 28 °C (301 K) to 50 °C (323 K)

Adsorbent	Temperature (K)	ΔG (kJ mol^–1^)	ΔH (kJ mol^–1^)	ΔS (J mol^–1^.K^–1^)
**NaZ**	301	– 3.785	+39.66	+144.16
	313	– 5.392		
	323	– 6.972		
**HZ**	301	5.890	+5.70	+16.89
	313	4.591		
	323	2.095		
**TD**	301	5.318	+21.79	+54.36
	313	5.037		
	323	4.079		

Based on the thermodynamic parameters, the overall adsorption process for the three adsorbents studied was spontaneous.^74–76^ It is also worth noting that the values of ΔG for the NaZ zeotype decreased with increasing temperature, indicating that the rise in temperature favored the adsorption mechanism. In contrast, for HZ and DE, the spontaneity of the MB adsorption process slightly reduced with the increase in temperature. This behavior has also been observed by Ji et al. (2021) e Li et al. (2022).

Table 11 presents the thermodynamic parameters for the adsorption of MB using NaZ, HZ, and DE at temperatures of 301, 313, 323, and 333 K.

The positive ΔH value for the process involving all adsorbents indicates that energy was absorbed during adsorption, providing clear evidence that the process was endothermic. In absolute terms, the ΔH values were below 40 kJ mol^–1^, suggesting that the reaction stage of sorption was of a physical nature, characterized by multilayer adsorption for all three solids evaluated, which corroborates the better fit to the Freundlich isotherm for NaZ and HZ.^75^

The highest adsorption heat (+39.66 kJ mol^–1^) was obtained with NaZ, indicating that the interaction between the MB molecule and the active sites was more effective, approaching energy release on the order of a chemical bond. In contrast, the heat released (enthalpy) for HZ was very low, akin to that of condensation, allowing the adsorbed molecule to move across the zeolitic surface. The enthalpy of adsorption for MB with DE was also low, suggesting that in this case, the adsorbed molecule on the diatomite surface did not bind to a specific site, facilitating the desorption process.

The change in entropy, represented by ΔS, showed positive values for the adsorption of MB on all surfaces. This indicates an increase in the degree of freedom or randomness at the solid–liquid interface during the solid–liquid process of MB. In other words, the number of molecules adsorbed on the surface is equivalent to the number of molecules diffusing from the solid medium to the liquid medium.^76,78^ This behavior was enhanced by the increased agitation and disorder of the molecules, driven by the rise in temperature. This increase in temperature also facilitated the greater diffusivity of MB molecules across the boundary layer of the external surfaces of the three adsorbents, as well as within the internal boundary layer of the NaZ zeotype.

Additionally, Gibbs free energy (Δ*G*) is a crucial parameter in adsorption studies. The data indicate that Δ*G* values were negative for (NaZ) and positive (with progressive decrease) for (HZ and DE) across the entire temperature range, suggesting that adsorption becomes increasingly favorable at higher temperatures. In contrast, the enthalpy (Δ*H*) and entropy (Δ*S*) parameters exhibited minimal sensitivity to temperature variations, allowing them to be considered practically constant under the investigated conditions. Given these characteristics, the adsorption process appears to be governed by entropic thermodynamic control. According to the fundamental Gibbs free energy equation, the entropy contribution (-TΔS) becomes more significant relative to the positive enthalpy values, leading to substantial variations in Δ*G*. As a result, an increase in temperature enhances the spontaneity of the process, driving a progressive decrease in Δ*G*.^79^

Comparing the ΔG results among the three adsorbents studied, it is evident that the most negative ΔG values were associated with NaZ, suggesting a more spontaneous and effective binding. In contrast, the less negative ΔG values were observed for HZ, which may be attributed to the higher acidity of B and L in this sample.^34,77^

## 4 Conclusions

In the present study, sodium (NaZ) and protonic (HZ) zeotypes synthesized from diatomaceous earth (DE) exhibited good adsorption capacity for the dye MB present in an aqueous solution. The adsorption process demonstrated rapid kinetics, with the best fit to the experimental data for the Avrami kinetic model and, the Freundlich isotherm for NaZ and, HZ, and Langmuir for DE. This suggests a physical-type surface interaction at the acidic sites of B and L with the MB molecule. According to the nF parameter of the Freundlich isotherm (ranging from 1 to 10), the adsorption mechanism is classified as favorable, while the thermodynamic analysis indicates that the process is spontaneous with increasing temperature for NaZ, and less spontaneous for HZ and DE. Through the study of kinetic models, equilibrium, and thermodynamics of adsorption, it was possible to elucidate the mechanisms governing the adsorption process on the solid surface of an adsorbent, using MB as a model molecule. In this context, the chemical and physical interactions between the adsorbents and the AM dye, discussed previously, demonstrate the high adsorption potential of the environmentally friendly materials, with an emphasis on the NaZ zeotype proposed in this study for the efficient removal of the dye in aqueous solution.

## Supplementary Material



## Notes

SEM micrographs related to diatomaceous earth and acidic and basic zeotypes­(PDF)
