# Supplementary material for: Insights into Kinetics and Thermodynamics for Adsorption Methylene Blue Using Ecofriendly Zeolites Materials
Source: ACS Omega. 2025 May 15;10(20):20326–40. doi: 10.1021/acsomega.4c11718 (PMC12120626; doi:10.1021/acsomega.4c11718)
Supplement: Supplementary file 1 [file ao4c11718_si_001.pdf]

Supporting Information

Insights into Kinetics and Thermodynamics for adsorption Methylene Blue using Ecofriendly Zeolites Materials

Mateus Gonçalves dos Santos[1*], *Lucas Destefani Paquini*[1,2], *Paulo Henrique Leite Quintela*[3], *Luciene Paula Roberto Profeti*[1] *and Damaris Guimarães*[1]

[1] Postgraduate program in Chemical Engineering (PPEQ), Universidade Federal do Espírito Santo, Alto Universitário, s/n., 29500-000 Alegre – ES, Brazil;

[2] Laboratório de Pesquisa e Desenvolvimento em Eletroquímica (LPDE), Universidade Federal do Espírito Santo, Campus Goiabeiras, Av. Fernando Ferrari, 29075-910 Vitória – ES, Brazil;

[3] Postgraduate program in Chemical Engineering, Universidade Federal de Sergipe (UFS), 49.100-000 São Cristóvão – SE, Brazil;

KEYWORDS: Sustainability. Wastewater treatment. Adsorption. Zeotypes.

*** Corresponding author**

Name: Mateus Gonçalves dos Santos

Telephone: (+55) 16 3351-8739

E-mail: mateus47@estudante.ufscar.br

The PDF file includes:

Figure S1. Micrographs obtained by scanning electron microscopy related to diatomaceous earth and acidic and basic zeotypes

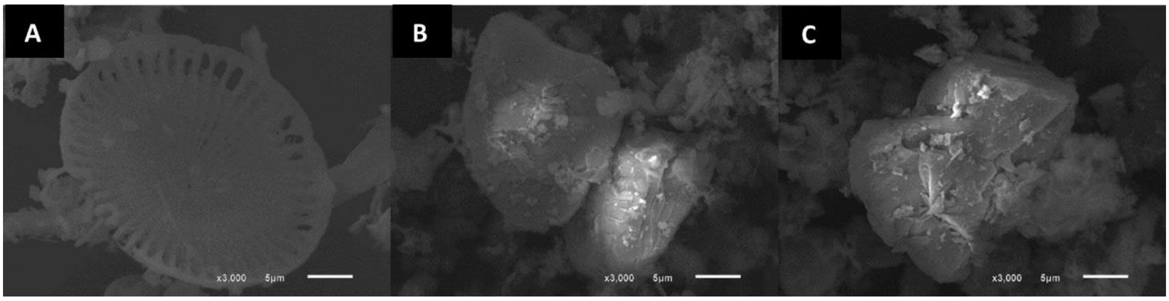

Figure S1. Scanning electron microscopy of the DE (A). NaZ (B) and HZ (C).
